# Diagnostic performance of clinic and home blood pressure measurements compared with ambulatory blood pressure: a systematic review and meta-analysis

**DOI:** 10.1186/s12872-020-01736-2

**Published:** 2020-11-23

**Authors:** Auttakiat Karnjanapiboonwong, Thunyarat Anothaisintawee, Usa Chaikledkaew, Charungthai Dejthevaporn, John Attia, Ammarin Thakkinstian

**Affiliations:** 1 Mahidol University Health Technology Assessment (MUHTA) Graduate Program, Mahidol University, Bangkok, Thailand; 2 Department of Family Medicine, Faculty of Medicine, Ramathibodi Hospital, Mahidol University, 270 Rama VI Road, Rachathevi, Bangkok, 10400 Thailand; 3 Division of Neurology, Department of Medicine, Faculty of Medicine, Ramathibodi Hospital, Mahidol University, Bangkok, Thailand; 4 School of Medicine and Public Health, University of Newcastle and Hunter Medical Research Institute, Newcastle, NSW Australia; 5 Department of Clinical Epidemiology and Biostatistics, Faculty of Medicine, Ramathibodi Hospital, Mahidol University, Bangkok, Thailand; 6 Social and Administrative Pharmacy Division, Department of Pharmacy, Faculty of Pharmacy, Mahidol University, Bangkok, Thailand

**Keywords:** Clinic blood pressure measurement, Home blood pressure measurement, diagnostic performance, Hypertension, Systematic review, Meta-analysis

## Abstract

**Background:**

Clinic blood pressure measurement (CBPM) is currently the most commonly used form of screening for hypertension, however it might have a problem detecting white coat hypertension (WCHT) and masked hypertension (MHT). Home blood pressure measurement (HBPM) may be an alternative, but its diagnostic performance is inconclusive relative to CBPM. Therefore, this systematic review aimed to estimate the performance of CBPM and HBPM compared with ambulatory blood pressure measurement(ABPM) and to pool prevalence of WCHT and MHT.

**Methods:**

Medline, Scopus, Cochrane Central Register of Controlled Trials and WHO's International Clinical Trials Registry Platform databases were searched up to 23rd January 2020. Studies having diagnostic tests as CBPM or HBPM with reference standard as ABPM, reporting sensitivity and specificity of both tests and/or proportion of WCHT or MHT were eligible. Diagnostic performance of CBPM and HBPM were pooled using bivariate mixed-effect regression model. Random effect model was applied to pool prevalence of WCHT and MHT.

**Results:**

Fifty-eight studies were eligible. Pooled sensitivity, specificity, and diagnostic odds ratio (DOR) of CBPM, when using 24-h ABPM as the reference standard, were 74% (95% CI: 65–82%), 79% (95% CI: 69%, 87%), and 11.11 (95% CI: 6.82, 14.20), respectively. Pooled prevalence of WCHT and MHT were 0.24 (95% CI 0.19, 0.29) and 0.29 (95% CI 0.20, 0.38). Pooled sensitivity, specificity, and DOR of HBPM were 71% (95% CI 61%, 80%), 82% (95% CI 77%, 87%), and 11.60 (95% CI 8.98, 15.13), respectively.

**Conclusions:**

Diagnostic performances of HBPM were slightly higher than CBPM. However, the prevalence of MHT was high in negative CBPM and some persons with normal HBPM had elevated BP from 24-h ABPM. Therefore, ABPM is still necessary for confirming the diagnosis of HT.

## Background

Screening for hypertension (HT) is an important strategy for prevention of cardiovascular diseases (CVD). Currently, several approaches are being used for measuring blood pressure (BP) including office or clinic blood pressure measurement (CBPM) and out-off office blood pressure measurement (i.e. ambulatory blood pressure measurement (ABPM) and home blood pressure measurement (HBPM)). HBPM is the average of all BP measurements performed by a semiautomatic BP monitor, for at least 3 days with readings in the morning and the evening, while ABPM records BP periodically at regular intervals (typically every 15, 20 or 30 min) for a pre-defined period of time [1].

Among all BP measurement methods, CBPM is the most commonly used in routine clinical practice, albeit there are two major concerns with CBPM. First, patients may have falsely high BP only in the clinical setting, i.e., a phenomenon known as white coat hypertension(WCHT), or they may have normal BP in the clinic but have an elevated BP measured by out-off office blood pressure measurement (i.e. ABPM or HBPM), known as masked hypertension (MHT) [2]. WCHT increased risk of cardiovascular diseases (CVD) about 19% when compared to normotension [3, 4], whereas MHT significantly increased risk of CVD about 3 times when compared to normotension [5]. The lack of recognition of WCHT and MHT results in patients with WCHT receiving unnecessary treatments and patients with MHT receiving delayed proper treatments. Therefore, accurate diagnosis of HT is crucial in preventing complications of HT and avoiding unnecessary treatment. Guidelines by European Society of Cardiology (ESC) and the European Society of Hypertension (ESH) 2018 [6], and American Heart Association (AHA) 2017 [2] suggest the use of out-off office blood pressure measurement (i.e. HBPM and/or ABPM) to confirm the diagnosis of HT as both ABPM and HBPM have the different advantages and disadvantages of identifying WCHT and MHT. HBPM is less expensive and more available than ABPM. However, HBPM do not measure BP during routine daily activities and during sleep. Thus, HBPM may have the potential of measurement error and incorrect classification of BP status, especially in persons having high nocturnal BP [1].

A meta-analysis conducted in 2011 [7] assessed the diagnostic performance of CBPM (N = 7) and HBPM (N = 3) using day-time ABPM as the reference standard. This meta-analysis found the overall sensitivity of CBPM to be lower than HBPM (74.6% vs. 85.7%), yet the specificity was higher (74.6% vs. 62.4%). Another systematic review in 2015 found that positive predictive values of CBPM (i.e. probability of being diagnosed with HT by ABPM or HBPM in persons with an elevated BP by CBPM) ranged from 35 to 95% [8]. However, this study did not apply meta-analysis to pool the diagnostic accuracy of CBPM.

Since 2011, there have been several published studies regarding CBPM and HBPM to date. New information regarding the factors associated with HT diagnosis such as age, sex, measurement technique, and types of ABPM have become available [9–11]. Performing a subgroup analysis on these factors may be useful in guiding BP screening strategies. Therefore, this systematic review was conducted with following aims: (1) to update the diagnostic performances of CBPM and HBPM using ABPM as the standard test and, (2) to pool prevalence of WCHT among positive CBPM, and pool prevalence of MHT among negative CBPM, (3) to perform subgroup analysis by those potential factors associated with HT diagnosis. The results derived from this study will have practical application for primary care, internal medicine physicians and cardiologists regarding the appropriate measurement method to use for HT diagnosis.

## Methods

The protocol of this systematic review has been registered in PROSPERO (CRD42018099647). The review protocol is available at https://www.crd.york.ac.uk/prospero/display_record.php?ID=CRD42018099647.

### Selection of studies

Relevant studies were identified from Medline, Scopus, Cochrane Central Register of Controlled Trials (CENTRAL) and WHO's International Clinical Trials Registry Platform (ICTRP) databases up to 23rd January 2020 using search terms and strategies described in the Additional file 1: Appendix. Reference lists of the included studies were searched to identify additional studies.

Study selection was manually performed by 2 independent reviewers (AK and TA). Studies were selected based on titles and abstracts and full articles were retrieved if more information was needed. The studies were eligible if (1) they included participants aged ≥ 18 years, (2) had diagnostic test as CBPM or HBPM with reference standard as ABPM, and (3) reported sensitivity and specificity and/or WCHT proportion among people with high BP from CBPM or MHT proportion among people with normal BP from CBPM. Endnote X9 was used to manage references during the process of study selection.

### Study and standard tests

The study tests were CBPM and HBPM. CBPM was performed in a health care setting, whereas HBPM was self-performed in a household using manual or automatic sphygmomanometers. The thresholds used for defining HT were BP ≥ 140/90 mmHg for CBPM and BP ≥ 135/85 mmHg for HBPM or as defined by the included studies [12–14]. The reference standard test was ABPM which measured BP daytime (10–16 h) or 24 h.

### Outcome of interest

The interested outcome was HT diagnosed by ABPM using the thresholds of ≥ 135/85, ≥ 120/70, and ≥ 130/80 mmHg for daytime, night-time, and 24-h, respectively or the thresholds as defined in the included studies. Diagnostic performance of studied tests (i.e., CBPM and HBPM) compared to the standard test (i.e. ABPM) was assessed by estimating sensitivity (i.e., probability of having positive CBPM among HT patients diagnosed by ABPM), specificity (i.e., probability of having negative CBPM among non-HT patients diagnosed by ABPM), likelihood ratio positive (LR + , i.e., sensitivity/(1-specificity)), likelihood ratio negative (LR-, i.e., (1-sensitivity)/specificity) and diagnostic odds ratio (DOR, i.e., LR + /LR-). WCHT was defined as normal BP measured by ABPM and/or HBPM among CBPM positive whereas MHT was defined as high BP measured by ABPM and/or HBPM among CBPM negative [15].

### Data extraction

Two reviewers (AK and TA) independently extracted the data including study’s characteristics (i.e. study setting, study design), study participants (i.e. mean age, percent male, and underlying disease), study and standard tests (i.e. types, measurement device, time and duration of measurement, and cut-offs for HT diagnosis). Numbers of true positive, false positive, true negative, and false negative for each diagnostic test were extracted.

### Risk of bias assessment

Risk of bias assessments were done independently by 2 reviewers (AK and TA) using the Quality of Diagnostic Accuracy Studies—2 (QUADAS-2) [16] including patient selection, index test, reference standard, and flow/timing domains. Each domain consists of two sections, i.e., risk of bias and applicability. Risk of bias comprised of 3 items (i.e., information used to support the risk of bias judgment, signaling questions and judgment) which was judged as low, high or unclear. Applicability was judged as low, unclear or high risk according to whether the study did or did not match the review question.

### Statistical analysis

Diagnostic performances of CBPM/HBPM versus ABPM (i.e., sensitivity, specificity, area under receiver operating characteristic (ROC) curve, LR^+^, LR^−^, and DOR were estimated for individual studies. These were then pooled using a bivariate mixed-effect regression model according to the types of ABPM and thresholds used for defining HT (i.e. 24-h ABPM with threshold of ≥ 130/80 mmHg, daytime ABPM with threshold of ≥ 135/85 mmHg). For studies that applied both 24-h and daytime ABPMs as the reference standards, only the data that used 24-h ABPM were used for pooling diagnostic performance of CBPM and HBPM. The hierarchic summary ROC(HSROC) curve was also estimated and plotted if applicable (number of studies ≥ 4); this was classified as low, moderate or high accuracy if the HSROCs were 0.5 < *x* < 0 .7, 0.7 ≤ *x* ≤ 0.9, and 0.9 < *x* ≤ 1, respectively [17].

Prevalence of WCHT and MHT were separately pooled using a random-effect model if heterogeneity was present; otherwise a fixed-effect model was applied. Heterogeneity was assessed using a Q test (p < 0.1) and the I^2^ statistic (> 25%). Potential sources of heterogeneity (i.e. results from risk of bias assessment, mean age, sex, study settings and numbers of repeated BP measurements) were explored by adding variables one by one in a meta-regression model. If the variables could decrease I^2^ or tau^2^, a subgroup analysis was performed accordingly.

Publication bias was examined by Deeks’ funnel plot [18]. If there was asymmetry, a contour-enhanced funnel plot was further explored to distinguish whether an asymmetrical funnel was due to heterogeneity or publication bias. All statistical analyses were performed with STATA version 15.0 (StataCorp, College Station, Texas). P-values < 0.05 were considered statistically significant for all tests with the exception of heterogeneity and Egger’s tests, where a P-value < 0.10 was used.

## Results

**Fig. 1:**
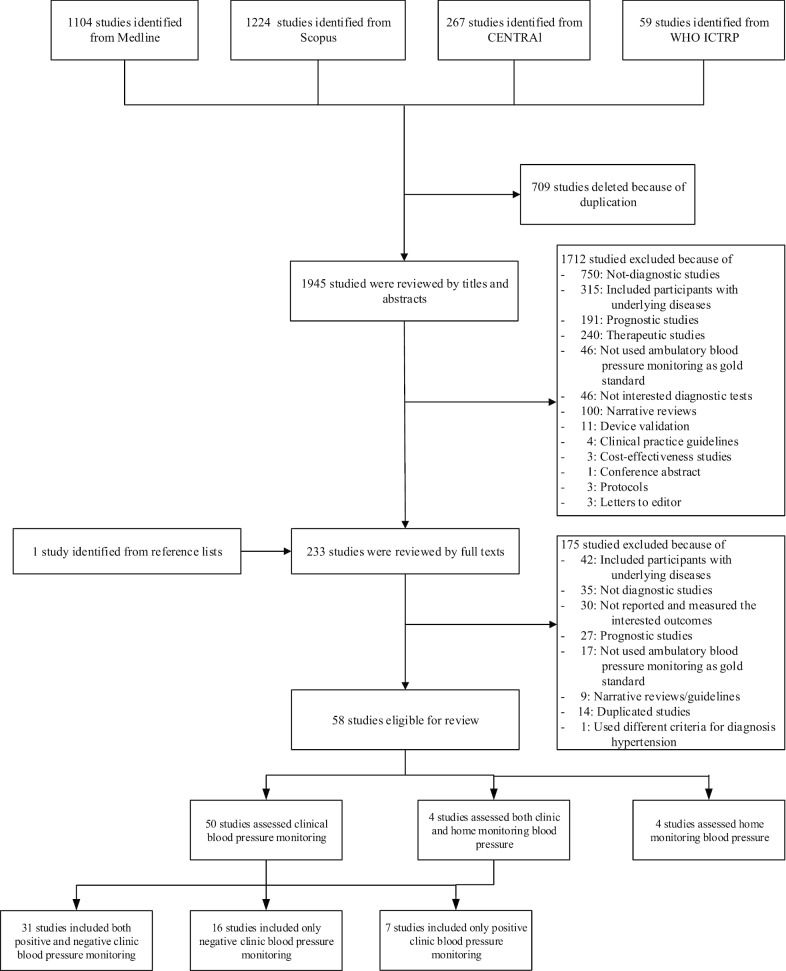
Flow chart of study selection

Searching from Medline, Scopus, CENTRAL and WHO ICTRP databases identified 1104, 1224, 267, and 59 articles, respectively. After deleting duplications, 1,945 studies were screened by titles and abstracts. A total of 233 full articles were reviewed. Fifty-eight studies met inclusion criteria and were included in the review. Among the included studies, 50 [9, 11, 19–66], 4 [67–70], and 4 [71–74] studies assessed CBPM, HBPM, and both CBPM and HBPM performances (see Fig. 1). Their characteristics are described in Table 1. Among the 58 studies, 32 and 26 studies recruited participants from hospital and community settings, correspondingly. Most studies included general population, whereas 4 studies included specific populations, i.e., white-collar workers [25, 66], male football players [40] and male military workers [37]. Twenty-one studies included participants who had not been prior diagnosed with HT, whereas 37 studies included both participant who had or had not been diagnosed with HT.

**Table 1:** Characteristic of included studies

Author (Year)	Country	Setting	Type of population	Previous HT	Taking anti-HT drug	Mean age (year)	%Male	CBPM; HBPM cut-off (mmHg)	No. of CBPM measurements per visit / No. of visit (interval)	No. of HBPM measurements per day/ No. of day	Time measuring ABPM	ABPM cut-off (mmHg)
*Studies with complete 2 × 2 diagnostic results of CBPM only*												
Brueren M. 1995 [19]	Netherlands	Health care	Outpatient	Non-HT	No	47	51.13	95(DBP)	1/3	–	Daytime (6 – 22.00)	91(DBP)
Botomino A. 2004 [20]	Switzerland	Community	General population	Mixed	Unspecified	53.7	42	140/90	2/1	–	Daytime (6 – 18.00)	135/85
Ungar A 2004 [21]	Italy	Health care	General population	Mixed	No	60	48.8	140/90	2/2 (1 day)	–	Daytime (7 – 22.00)	135/85
Ohkubo T. 2005 [22]	Japan	Community	General population	Mixed	Mixed	61	40	140/90	2/1	–	Daytime	135/85
Fagard R 2007 [23]	EU-countries	Community	General population	Mixed	No	39	46.1	140/90	3/1	–	Daytime (8 – 22.00)	135/85
Wang G 2007 [24]	China	Community	General population	Mixed	Mixed	(18 – 86)	54.3	140/90	5/1	–	Daytime (8 – 18.00)	135/85
Trudel X 2009 [25]	Canada	Community	White-collar workers	Non-HT	No	44	38.8	140/90	3/1	–	Daytime	135/85
Ishikawa J. 2010 [26]	Japan	Community	General population	Mixed	No	59.9	52.7	140/90	2/1	–	24-h	130/80
Maseko M 2011 [27]	South Africa	Community	General population	Mixed	Unspecified	43.9	35.6	140/90	5/1	–	Daytime (9 – 19.00)	140/85
Afsar B. 2013 [28]	Turkey	Health care	Outpatient	Mixed	Unspecified	57.46	67	140/90	2/1	–	Daytime (7 – 23.00)	135/85
Berge H 2013 [40]	Norway	Community	Male professional football players	Mixed	No medication	28.1	–	140/90	2/1	–	Daytime (7 – 22.00)	135/85
Alwan H. 2014 [29]	Germany	Community	General population	Mixed	Mixed	48	49	140/90	5/1	–	Daytime (7 – 22.00)	135/85
Conen D. 2014 [9]	Europe, South America and Asia	Community	General population	Mixed	No	45.74	48.48	140/90	2/1	–	Daytime Europe/America:10–20.00 Asian:8–18.00	135/85
											24-h	130/80
Al-Hashimi K 2015 [30]	Oman	Health care	Outpatient	Mixed	Mixed	46.15	–	140/90	1/1	–	Daytime	135/85
Rhee M 2015 [11]	Korea	Community	General population	Mixed	Unspecified	46.7	35.9	140/90	3/1	–	24-h	130/80
Mutlu S. 2016 [31]	Turkey	Health care	General in-patient	Mixed	Mixed	44.4	54.4	140/90	1/1	–	24-h	125 –130/80
Scuteri A. 2016 [32]	Italy	Community	General population	Mixed	Mixed	48.93	39.88	140/90	NR	–	24-h	125/79
Melgarejo J 2017 [33]	Europe and Asia	Community	General population	Mixed	No	–	–	140/90	3/1	–	24-h	130/80
Fujita H. 2018 [34]	Japan	Community	General population	Non-HT	No	–	51.5	140/90	3/1	–	Daytime (8 – 20.00)	135/85
Erdogmus S. 2018 [61]	Turkey	Health care	General population	Mixed	Mixed	55	42.3	140/90	3/1	–	24-h	130/80
Sheppard J.P. 2018 [65]	U.K	Health care	General population	Mixed	Mixed	52.8	46.2	NR	1/1	–	Daytime	135/85
Bhattarai M. 2019 [58]	Nepal	Health care	General population	Non-HT	Mixed	43.82	55	140/90	3/1	–	24-h	130/80
Cai P. 2019 [59]	China	Health care	General population	Non-HT	No	61.8	48.5	140/90	3/1	–	24-h	130/80
Kaul U. 2019 [62]	India	Health care	General population	Mixed	Mixed	NA	NA	140/90	1/1	–	24-h	130/80
Michaud A. 2019 [63]	Canada	Health care	Outpatient	Mixed	Unspecified	51.9	54	140/90	1/1	–	24-h	130/80
Trudel X. 2020 [66]	Canada	Community	Worker	Mixed	Mixed	44.6	40.2	140/90	3/1	–	Daytime (8–16.00)	135/85
Calvo-Vargus C. 2003 [60]	Mexico	Health care	General population	Mixed	Mixed	54.7	82.2	140/90	3/2 (14 days)	–	Daytime (6–22.0)	135/85
*Studies with complete 2 × 2 diagnostic results of HBPM only*												
Almeida A. 2012 [67]	Brazil	Health care	General population	Mixed	Mixed	50.6	–	135/85	–	12/5	24-h	130/80
											Daytime	135/85
Park J. 2017 [68]	Korea	Health care	Outpatient	Mixed	Unspecified	51.8	46.5	135/85	–	3/6	24-h	130/80
de Almeida, 2014 [69]	Brazil	Health care	Outpatient	Mixed	Mixed	50.6	46.8	135/85	–	12/3	Daytime	135/85
Rhee M.Y., 2018 [70]	South Korea	Health care	General population	Non-HT	No	52.2	47.1	135/85	–	3/7	24-h	130/80
*Studies with complete 2 × 2 diagnostic results of both CBPM and HBPM*												
Stergiou G 2000 [73]	Greece	Health care	Outpatient	Mixed	No	48.4	54.9	140/90; 140/90	3/5 (14 – 21 days)	2/6	Daytime	140/90
Hanninen M 2010 [72]	Finland	Community	General population	Mixed	Mixed	49.1	47.9	140/90; 135/85	2/4 (21 days)	2/7	Daytime (6 – 23.00)	140/85
Zhang L 2015 [71]	Belgium	Health care	General population	Mixed	No medication or wash out period > 2 weeks	50.6	51.1	140 (SBP); 135/85	3/3 (7 days)	6/7	24-h	130/80
Ntineri A. 2019 [74]	Greece, U.K., Finland	Health care	General population	Mixed	Mixed	53.8	52.6	140/90: 135/85	3/3 (10–21 days)	2/7	24-h	130/80
*Studies with positive CBPM studies only*												
Hoegholm A. 1999 [35]	Denmark	Community	General population	Mixed	No medication or wash out period > 5 weeks	47.7	46.9	140/90	3/3 (7 days)	–	Daytime (7 – 23.00)	135/85
Martinez M 1999 [36]	Spain	Health care	General population	Non-HT	No medication or wash out period > 3 weeks	52	47.8	140/90	2/3 (7–14 days)	–	Daytime (10 – 20.00)	135/85
Gan S 2003 [37]	Singapore	Community	Male military conscripts	Mixed	Unspecified	20	100	140/90	2/2 (14 days)	–	24-h	135/85
Tunckale A. 2004 [38]	Turkey	Health care	General patient	Non-HT	No medication or wash out period > 4 weeks	–	–	140/90	1/3	–	Daytime (6 – 24.00)	135/85
Shimbo D. 2009 [39]	U.S.A	Health care	Outpatient	Mixed	No medication	52.5	46	140/90	3/2 (30 days)	–	Daytime (6 – 22.00)	135/85
Pengkeaw P. 2014 [41]	Thailand	Health care	Outpatient	Mixed	No medication or wash out period > 4 weeks	42.29	74.2	140/90	2/1	–	Daytime	135/85
Mancia G.2015 [42]	Italy	Community	General population	Unspecified		–	–	140/90	3/2	–	24-h	130/80
*Studies with negative CBPM only*												
Schoenthaler A 2010 [43]	U.S. (African or Latino)	Community	General population	Mixed	No medication	35.9	39	140/90	3/3 (14 days)	–	Daytime	135/85
Viera A 2010 [44]	U.S	Community	General population	Non-HT	No medication	49	44	140/90	3/1	–	Daytime (6- 23.00)	135/85
											24-h	130/80
Bacaksiz A. 2013 [45]	Turkey	Health care	Outpatient	Non-HT	No medication	35.8	51.1	140/90	2/1	–	Daytime (9–21.00)	135/85
Sobrino J. 2013 [46]	Spain	Community	General population	Non-HT	No medication	43.1	44.7	140/90	3/1	–	24-h	130/80
											Daytime	135/85
Franklin S 2013 [47]	Europe Japan	Community	General population	Non-HT	Mixed	–	–	140/90	2/1	–	Daytime	135/85
Larsen T 2014 [48]	America	Health care	Outpatient	Non-HT	No medication	49.8	47	140/90	1/3	–	24-h	135/85
Viera A 2015 [49]	America	Health care	Outpatient	Non-HT	No medication	47	39	140/90	3/1	–	24-h	130/80
Trachsel L 2015 [50]	Switzerland	Community	General population	Non-HT	No medication	42.8	–	140/90	3/1	–	24-h	130/80
Redmond N. 2016 [51]	America,	Community	General population	Mixed	Mixed	59.1	30.7	140/90	2/1	–	Daytime (10 – 20.00)	135/85
Viera A 2016 [52]	America	Health care	General population	Non-HT	No medication	48	40	140/90	3/1	–	Daytime	135/85
Piantanida. E 2016 [53]	Italy	Health care	General population	Non-HT	No medication	46.3	–	140/90	3/1	–	24-h	130/80
Booth III J 2017 [54]	America	Health care	Outpatient	Non-HT	No medication	–		140/90	2/1	–	Daytime	135/85
Anstey D 2017 [55]	America,	Community	General population	Mixed	Mixed	56	35.7	140/90	2/1	–	Daytime (10–20.00)	135/85
Ozkan S. 2018 [56]	Turkey	Health care	Outpatient	Non-HT	No medication	58.8	25.1	140/90	NR	–	24-h	130/80
Gun T. 2018 [57]	Turkey	Health care	Outpatient	Non-HT	No medication	55	46.5	140/90	1/1	–	24-h	130/80
Salazar M. R., 2019 [64]	Argentina	Health care	Outpatient	Mixed	Mixed	51.4	40.7	140/90	3/1	–	24-h	Day:135/85 Night:120/70

*ABPM* ambulatory blood pressure measurement, *CBPM* clinic blood pressure measurement, *DBP* diastolic blood pressure, *HBPM* home blood pressure measurement, *HT* hypertension, *NR* not reported, *SBP* systolic blood pressure

### Risk of bias assessment

Results of risk of bias assessment are presented in Additional file 1: Table 1. Almost all CBPM studies (94.44%) were low risk in all domains of applicability. Eight [20, 31, 38, 39, 44, 50, 52, 57] (16.7%) and 7 (12.9%) studies [30, 34, 41, 46, 49, 55, 58] were high or unclear bias in selection of study subjects, accordingly. Fifty-two studies (96.3%) [9, 11, 19–31, 33–38, 40–66, 70, 71, 74] applied the index/study test before the reference standard but with unclear explanation of blinding. Thirty-eight studies (70.4%) were high or unclear risk of bias in flows and timing. This is due to a lack of reporting the time interval between the study test and the reference standard or the exclusion of subjects with invalid test results or those lost to follow up. All HBPM studies were low risk of bias in all domains of applicability. Six studies (75%) applied the index test before the reference standard without blinding information, and 4 studies (50%) were high risk of bias in their flow and timing.

### Pooling CBPM diagnostic performances

Among 54 CBPM studies, 31 studies [9, 11, 19–34, 40, 58–66, 71–73] reported 2 × 2 table data which could be assessed for diagnostic performance, while 7 [35–39, 41, 42] and 16 [43–57, 70] studies reported data for only positive and negative CBPM respectively (see Table 1). The mean age ranged from 28 to 62 years and percent male ranged from 35 to 100%. The number of CBPM measurements per visit ranged from 1 to 5 times (Table 1).

**Fig. 2:**
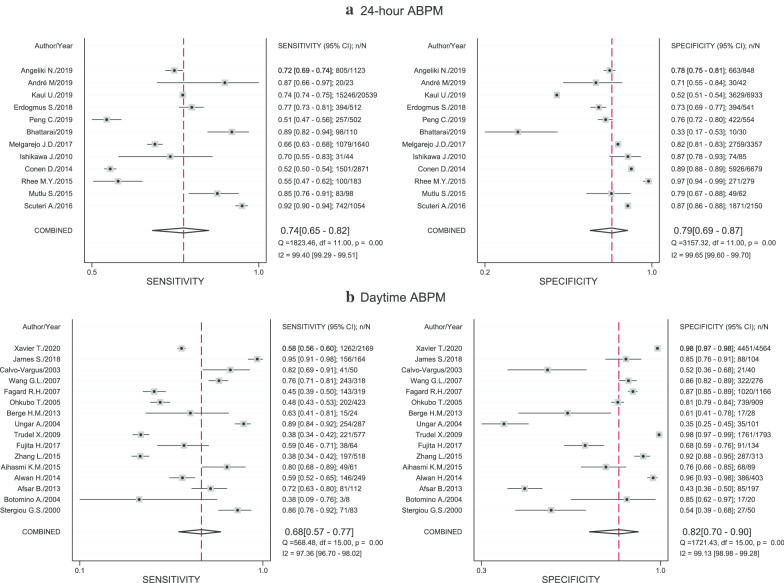
Pooled sensitivity and specificity of clinic blood pressure measurements compared with 24-h and daytime ambulatory blood pressure measurements. “n” referred to number of hypertensive patients who had positive clinic blood pressure measurement and number of non-hypertensive patients who had negative clinic blood pressure measurement for pooling sensitivity and specificity, respectively. “N” referred to number of hypertensive patients and non-hypertensive patients for pooling sensitivity and specificity, respectively. Reference line referred to pooled sensitivity or pooled specificity

**Fig. 3:**
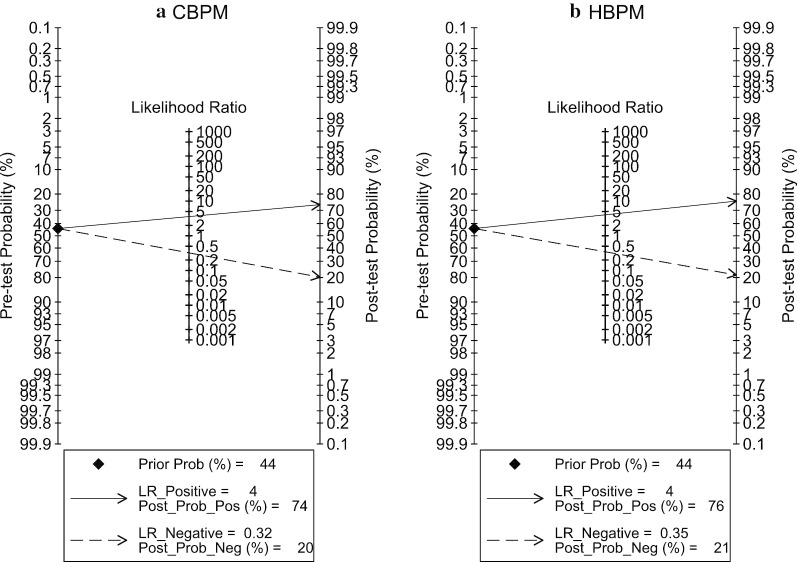
Fagan’s plot of clinic and home blood pressure measurements compared with 24-h ambulatory blood pressure measurement

Among the studies that reported 2 × 2 data (n = 66,767), 29 studies [9, 11, 20–34, 40, 58–63, 66, 72–74] used a CBPM cutoff threshold as ≥ 140/90 for diagnosis of HT, while one study [19] used the threshold of DBP > 95 mmHg and one study did not reported the threshold. The 24-h ABPM had a cut-off of ≥ 130/80 mmHg for 12 studies [11, 26, 31–33, 58, 59, 61–63, 74] and daytime ABPM had a cut-off of ≥ 135/85 mmHg for 16 studies [20–25, 28–30, 34, 60, 65, 66, 73]. Two [27, 72] and one [19] studies applied daytime ABPM with cut-offs of ≥ 140/85 and DBP ≥ 95 mmHg, respectively (see Additional file 1: Table 2). When using the 24-h ABPM with the cut-off of ≥ 130/80 mmHg as the reference standard, the diagnostic performance of CBPM were 0.74 (95% CI 0.65–0.82; I^2^ = 99.4%), 0.79 (95% CI 0.69, 0.87; I^2^ = 99.65%), 3.6 (95% CI 2.4, 5.3; I^2^ = 99.67%) and 0.32 (95% CI 0.24, 0.44; I^2^ = 99.58%) for sensitivity, specificity, LR + and LR-, respectively (see Fig. 2a and Additional file 1: Fig. 1). These diagnostic characteristics all require setting a threshold and trading off sensitivity for specificity or LR + for LR- hence they must be judged in pairs. For example, given a pretest probability of HT of 44%, the post-test probability was increased to 74% if CBPM was positive or reduced to 20% if CBPM was negative (see Fagan’s plot Fig. 3a). Alternatively, a single measure of diagnostic performance, i.e., the DOR was 11.11 (95% CI 6.44, 19.160; I^2^ = 100%), see Additional file 1:Fig. 1c. The HSROC reflects diagnostic performance across the entire range of possible threshold values; in this case, the pooled HSROC was 0.83 (95% CI 0.82, 0.85) indicating moderately good discrimination for judging presence of HT (Additional file 1: Fig. 2a).

When using daytime ABPM with cut-off of 135/85 mmHg as the reference standard, the pooled sensitivity and specificity were 68% (95% CI 57, 77; I^2^ = 97.36%) and 82% (95% CI 70, 90; I^2^ = 99.13%), see Fig. 2b. In addition, LR + , LR- and DOR of CBPM were 3.7 (95% CI 2.3, 6.0; I^2^ = 98.57%), 0.39 (95% CI 0.30, 0.52; I^2^ = 94.54%) and 9.46 (95% CI 5.39, 16.60; I^2^ = 100%), accordingly (see Additional file 1: Fig. 3). When all ABPMs with no restriction on the cutoffs as the reference standards were used, the pooled sensitivity and specificity were 70% (95% CI 63%, 76%; I^2^ = 98.56%) and 81% (95% CI 73%, 87%; I^2^ = 99.47%) and pooled LR + and LR- were 3.67 (95% CI 2.69, 5.00; I^2^ = 99.35%) and 0.37 (95% CI 0.31–0.44; I^2^ = 98.57%). For publication bias, Deeks’ funnel plot showed no evidence of publication bias (Additional file 1: Fig. 4a).

**Fig. 4:**
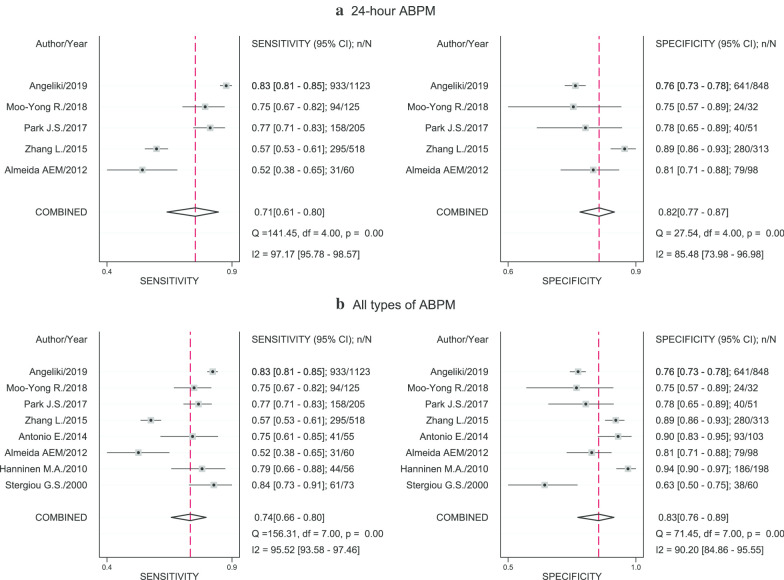
Pooled sensitivity and specificity of home blood pressure measurements compared with 24-h and all types ambulatory blood pressure measurements. “n” referred to number of hypertensive patients who had positive home blood pressure measurement and number of non-hypertensive patients who had negative home blood pressure measurement for pooling sensitivity and specificity, respectively. “N” referred to number of hypertensive patients and non-hypertensive patients for pooling sensitivity and specificity, respectively. Reference line referred to pooled sensitivity or pooled specificity

### Subgroup analysis

Subgroup analyses were performed by results of risk of bias assessment, age group (< 50 and ≥ 50 years), percent males (< 50% and ≥ 50%), number of repeated measurements of CBPM (1, 2–5 times), setting of studies (community and hospital-based) and type of patients (no HT, mixed HT with non-HT). When considering only studies with low risk of bias in the domain of flow and timing (N = 7), pooled sensitivity, specificity, LR + and DOR of CBPM were 73% (95% CI 60, 83), 75% (95% CI 51, 89), 2.9 (95% CI 1.5, 5.3), and 8 (95% CI 5, 14), respectively. The degrees of heterogeneity (I^2^) did not decrease for each sub-group of these factors (Additional file 1: Table 3), but performances of CBPM improved in some sub-groups including age group ≤ 50 year, percent male ≤ 50% and community-based setting with the LR + of 5.1 (95% CI 3.0, 8.7), 5.8 (95% CI 3.5, 9.8), and 6.0 (95% CI 3.9, 9.3), respectively.

### Pooling HBPM diagnostic performances

Eight HBPM studies [67–74] reported 2 × 2 data (Additional file 1: Table 4) with cutoff threshold of 135/85 [67–71, 74] (N = 7) and 140/90 [73] (N = 1) mmHg and measurement duration of about 3 to 7 days. The number of measurements per day ranged from 2 to 12 times (see Table 1). Mean age and percent male ranged from 48.1 to 51.8 years and 46.5% to 54.9% respectively. Among them, five and three studies applied 24-h and daytime ABPM, respectively.

The pooled sensitivity, specificity, DOR, LR + and LR- of HBPM were respectively 0.71 (95% CI 0.61, 080; I^2^ = 97.17%), 0.82 (95% CI 0.77, 0.87; I^2^ = 85.48), 11.60 (95% CI 8.98, 15.13; I^2^ = 100%), 4.02 (95% CI 3.38, 4.78; I^2^ = 19.39%) and 0.35 (95% CI 0.26, 0.46; I^2^ = 95.69%), when the 24-h ABPM with cut-off of ≥ 130/80 mmHg was applied as the reference standard (see Fig. 4a and Additional file 1: Fig. 5). In addition, among persons having HBPM positive, 14% had normotension from 24-h ABPM. In contrast, 40% of those having negative HBPM were diagnosed with hypertension from 24-h ABPM. Again, given a pretest-probability of 44%, a positive HBPM would result in a post-test probability of 76%, while a negative HBPM would reduce the probability to 21% (Fig. 3b). The pooled HSROC was 0.85 (95% CI 0.82, 0.88), reiterating moderately good discrimination, see Additional file 1: Fig. 2b.

**Fig. 5:**
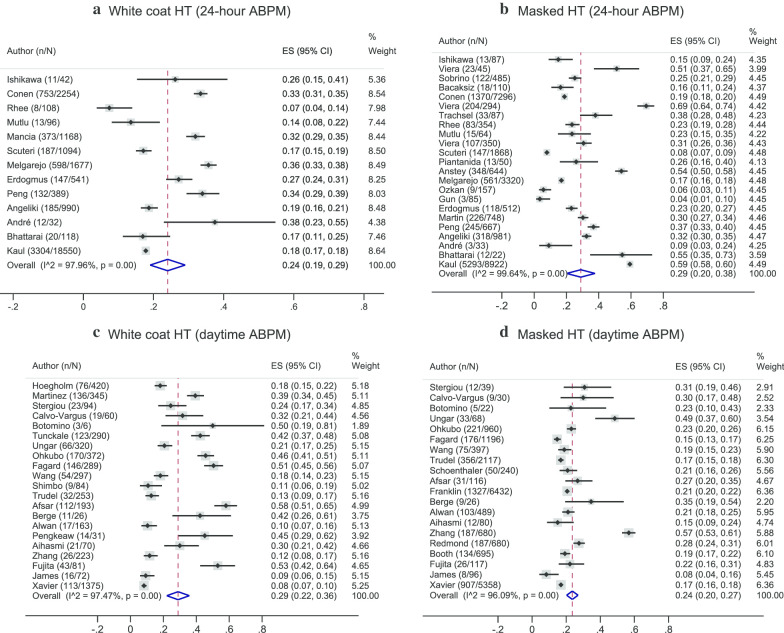
Pooled prevalence of white coat hypertension and masked hypertension using daytime and 24-h ambulatory blood pressure measurements as the reference standards. “n” referred to number of false positive and false negative clinic blood pressure measurements for pooling prevalence of white coat and masked hypertension, respectively. “N” referred to number of positive and negative clinic blood pressure measurements for pooling prevalence of white coat and masked hypertension, respectively. Reference line referred to pooled prevalence of white coat or masked hypertension

When all types ABPM with all cut-offs as the reference standard were applied, the overall pooled sensitivity and specificity were respectively 74% (95% CI 66%, 80%; I^2^ = 95.52%) and 83% (95% CI 76%, 89%; I^2^ = 90.20%), see Fig. 4b. The pooled DOR, LR + and LR-were 13.73 (95% CI 8.55, 22.03; I^2^ = 99.99%), 4.36 (95% CI 3.04, 6.27; I^2^ = 75.06%), and 0.32 (95% CI 0.25, 0.41; I^2^ = 94.34%), respectively. Analysis using daytime ABPM as the reference standard and subgroup analysis of HBPM could not be performed due to the small number of included studies. Deeks’ funnel plot indicated no evidence of publication bias, see Additional file 1: Fig. 4b.

### Pooling prevalence of WCHT and MHT by CBPM

Seven [35–39, 41, 42] and 16 studies [43–57, 64] reported only data of WCHT and MHT, see Table 1. These studies were then combined with 31 CBPM studies with 2 × 2 data above yielding a total of 38 and 47 studies for pooling proportions of WCHT and MHT, respectively. Among the 38 studies with WCHT, time of ABPM measures were 24-h (N = 14) and daytime (N = 24). Among the 47 studies with MHT, ABPM measurements were 24-h ABPM (N = 20) and daytime ABPM (N = 27). Four studies compared the performance of CBPM with both HBPM and ABPM but only two studies provided the number of people who had negative CBPM but had high blood pressure from either ABPM or HBPM. Therefore, most studies (N = 45) used only ABPM as the reference standard for pooling the prevalence of MHT.

Using the 24-h ABPM with a cut-off of 130/80 mmHg as the reference standard (N = 23), the pooled prevalence of WCHT and MHT were 0.24 (95% CI 0.19, 0.29; I^2^ = 97.96%) and 0.29 (95% CI 0.20, 0.38; I^2^ = 99.64%), see Fig. 5a and 5b. If daytime ABPM with cut-off of 135/85 mmHg was applied as the reference standard, the pooled prevalence of WCHT (N = 21) and MHT (N = 20) would be 0.29 (95% CI 0.22, 0.36; I^2^ = 97.47%) and 0.24 (95% CI 0.20, 0.27; I^2^ = 96.09%), see Fig. 5c and 5d.

When all types of ABPM were applied with any cut-offs as the reference standard, the pooled prevalence of WCHT (N = 38; n = 32,685) and MHT (N = 47; n = 47,713) were 0.28 (95% CI 0.25, 0.32) and 0.27 (95% CI 0.22, 0.32). Subgroup analyses were performed, but none of the co-variables could decrease the degree of heterogeneity (see Additional file 1: Table 7). However, subgroup of repeated measures of CBPM 4–5 times and 24-h ABPM could respectively reduce the pooled WCHT from 0.28 to 0.23 (95% CI 0.16, 0.31) and 0.23 (95% CI 0.18, 0.28). Likewise, repeated CBPM measure could reduce the pooled MHT from 0.27 to 0.15 (95% CI 0.10, 0.19) whereas the 24-h ABPM conversely increased the prevalence to 0.33 (95% CI 0.22, 0.43).

## Discussion

The findings suggest that when using 24-h ABPM as the reference standard, diagnostic performances of HBPM were slightly higher than those of CBPM. The pooled sensitivity, specificity, DOR, LR + and area under ROC for HBPM were respectively 71%, 82%, 11.60, 4.02 and 0.85, while these corresponding values for CBPM were respectively 74%, 79%, 11.11, 3.6, and 0.83.

To date, there has been only one relevant meta-analysis published in 2011 [7], which included fewer studies than ours (i.e., 7 versus 31 for CBPM and 3 versus 8 for HBPM). Overall sensitivities found in our study were lower than the previous review (i.e., 74% vs. 75% for CBPM and 71% vs. 86% for HBPM), while the specificities were higher (i.e., 79% vs. 75% for CBPM and 82% vs. 62% for HBPM). Our pooled estimates are more precise than the previous review which was limited by the small number of included studies. In addition, our results indicated that roughly 29% of those who are positive on CBPM may have WCHT and roughly 24% of those who are negative on CBPM may have MHT, when using daytime ABPM as the reference standard.

However, when using 24-h ABPM as the reference standard, the percent of people having WCHT reduced from 29 to 24%, while the percent of people having MHT increased from 24 to 29%. This reinforces the belief that 24-h ABPM yields the best detection for HT because it can capture the nighttime and morning surge BP. The number of repeated measurements of CBPM also affected the diagnostic performance, i.e., there was a lower WCHT and MHT if repeatedly measuring CBPMs over 4–5 visits.

The misclassification of patients who actually do not have hypertension is an important issue for diagnosis and treatment of hypertension because previous evidence from meta-analyses found a similar risk of cardiovascular disease between those with WCHT and normotension [75–77]. Unnecessary treatments of WCHT have several disadvantages including potential adverse drug events and costs. Measuring BP in the patient’s own environment using HBPM could reduce stress and decrease over-diagnosis of HT.

In contrast to WCHT, detection of MHT is important for CVD prevention. Our results found that prevalence of MHT was high in normal CBPM (29%). Even in people with negative HBPM, 40% of them had high BP when performing 24-h ABPM. Thus, ABPM is still necessary for confirming the diagnosis of MHT. Nonetheless, screening all individuals with normal CBPM is impractical; so prioritizing people who are high risk of CVD to screen with ABPM is important. According to the 2018 ESC/ESH guideline, persons with high normal office BP or with normal office BP but having hypertension-mediated organ damage or at high total cardiovascular risk are indicated for ABPM or HBPM monitoring [1].

Our study has some strengths. We estimated the diagnostic performance of CBPM and HBPM relative to ABPM by additionally pooling data from 31 and 8 studies with prevalence of WCHT and MHT. However, our study also faced limitations. Firstly, our pooling was based on high heterogeneity across studies, particularly for pooling prevalence of WCHT and MHT. Although we attempted to explore the sources of heterogeneity by performing subgroup analyses according to age group, sex, and results from risk of bias assessment, none of them was identified as a source of heterogeneity. A small number of HBPM studies was available compared to the large number of CBPM studies, and estimation of diagnostic performance yielded imprecision. Thus, results need to be updated when more studies are available, or individual patient data (IPD) meta-analysis should be considered to allow for more sub-group analysis of specific factors. Although most included studies had low risk of bias in subject selection and index test, most of them (70.4% for CBPM and 50% for HBPM) had high and unclear risk of bias in flow and timing due to long/unclear time interval between performing index and standard tests.

The long interval may lead to misclassification of disease due to improvement or worsening of the BP condition [16]. For instance, patients with high BP by CBPM/HBPM may be prescribed anti-hypertensive drugs to lowering BP before performing ABPM. This might underestimate the diagnostic performance of CBPM and HBPM. Finally, we did not identify relevant studies from grey/unpublished databases. Although there was no evidence of publication bias suggested by Deeks’ funnel plot [18] for both pooled estimates of CBPM and HBPM, potential publication bias could not be ruled out and overestimated diagnostic accuracy of CBPM and HBPM might be present. However, some previous systematic review and meta-analyses found that including unpublished studies might have a minimal effect on the overall estimates, so they should not impact the overall findings [78, 79].

## Conclusion

In conclusion, diagnostic performances of HBPM were slightly higher than the performance of CBPM. However, the prevalence of MHT was high in negative CBPM and some persons with normal HBPM had elevated BP from 24-h ABPM. Therefore, ABPM is still necessary for confirming the diagnosis of HT, especially in people who have high normal CBPM/HBPM or normal CBPM/HBPM with hypertension-mediated organ damage or at high CVD risk.

## Supplementary information

**Additional file 1**. Additional appendix, tables, and figures.

## Data Availability

All data generated or analysed during this study are included in this published article and its Additional information files.

## Abbreviations

ABPM: Ambulatory blood pressure measurement
AHA: American Heart Association
BP: Blood pressure
CBPM: Clinic blood pressure measurement
CI: Confidence interval
CKD: Chronic kidney disease
CVD: Cardiovascular diseases
DBP: Diastolic blood pressure
DM: Diabetes mellitus
DOR: Diagnostic odds ratio
HBPM: Home blood pressure measurement
HT: Hypertension
LR: Likelihood ratio
MHT: Masked hypertension
NM: Normotension
QUADAS-2: Quality of Diagnostic Accuracy Studies-2
ROC: Receiver operating characteristic
SBP: Systolic blood pressure
SD: Standard deviation
SE: Standard error
WCHT: White coat hypertension
