# Supplementary material for: Diagnostic performance of clinic and home blood pressure measurements compared with ambulatory blood pressure: a systematic review and meta-analysis
Source: BMC Cardiovasc Disord. 2020 Nov 23;20:491. doi: 10.1186/s12872-020-01736-2 (PMC7681982; doi:10.1186/s12872-020-01736-2)
Supplement: Supplementary file 1 — Additional file 1. Additional appendix, tables, and figures. [file 12872_2020_1736_MOESM1_ESM.docx]

**Title:** Diagnostic performance of clinic and home blood pressure measurements compared with ambulatory blood pressure: a systematic review and meta-analysis

Auttakiat Karnjanapiboonwong, M.D.

Health Technology Assessment (HTA) Postgraduate Program, Mahidol University, Bangkok, Thailand, Email: [auttakiat@yahoo.com](mailto:auttakiat@yahoo.com)

Thunyarat Anothaisintawee*, M.D., Ph.D.

Department of Family Medicine, Faculty of Medicine, Ramathibodi Hospital, Mahidol University, Bangkok, Thailand, Email: [thunyarat.ano@mahidol.ac.th](mailto:thunyarat.ano@mahidol.ac.th)

Usa Chaikledkaew, B.Pharm, Ph.D.

Health Technology Assessment (HTA) Postgraduate Program, Mahidol University, Bangkok, Thailand, Email: [usa.cha@mahidol.ac.th](mailto:usa.cha@mahidol.ac.th)

Charungthai Dejthevaporn, M.D., Ph.D.

Division of Neurology, Department of Medicine, Faculty of Medicine, Ramathibodi Hospital, Mahidol University, Bangkok, Thailand, Email: [charungthaid@gmail.com](mailto:charungthaid@gmail.com)

John Attia, M.D., Ph.D.

School of Medicine and Public Health, University of Newcastle and Hunter Medical Research Institute, Newcastle, New Southwale, Australia, Email: john.attaia@newcastle.edu.au

Ammarin Thakkinstian, Ph.D.

Section for Clinical Epidemiology and Biostatistics, Faculty of medicine, Ramathibodi Hospital, Mahidol University, Bangkok Email: [ammarin.tha@mahidol.ac.th](mailto:ammarin.tha@mahidol.ac.th)

**Additional Appendix**

**The search terms and search strategy used for Medline database**

((((((((((("clinic blood pressure") OR "office blood pressure") OR "conventional blood pressure") OR "clinic BP measurement") OR CBPM) OR "conventional BP monitoring") OR "clinic BP monitoring")) OR ((((("home blood pressure") OR "home monitoring blood pressure") OR "home BP measurement") OR "HBPM") OR "home BP monitoring")))) AND (((((((((((((((((sensitivity) OR specificity) OR "false positive") OR "false negative") OR "true positive") OR "true negative") OR "diagnostic performance"))) OR ((((("white coat") OR "white-coat") OR isolate* AND clinic) OR isolate* AND office) OR "mask hypertension"))) OR "masked hypertension"))))))

**The search term and search strategy used for Scopus database**

( ( ( TITLE-ABS-KEY ( "Office blood pressure" ) ) OR ( TITLE-ABS-KEY ( "Clinic blood pressure" ) ) OR ( TITLE-ABS-KEY ( "Home blood pressure" ) ) OR ( TITLE-ABS-KEY ( "Home monitoring blood pressure" ) ) ) OR ( TITLE-ABS-KEY ( "conventional blood pressure" ) ) OR ( TITLE-ABS-KEY ( "clinic BP measurement" ) ) OR ( TITLE-ABS-KEY ( cbpm ) ) OR ( TITLE-ABS-KEY ( "conventional BP monitoring" ) ) OR ( TITLE-ABS-KEY ( "clinic BP monitoring" ) ) OR ( TITLE-ABS-KEY ( "home BP measurement" ) ) OR ( TITLE-ABS-KEY ( "home BP monitoring" ) ) OR ( TITLE-ABS-KEY ( hbpm ) ) ) AND ( ( ( TITLE-ABS-KEY ( "true positive" ) ) OR ( TITLE-ABS-KEY ( "true negative" ) ) OR ( TITLE-ABS-KEY ( "false negative" ) ) OR ( TITLE-ABS-KEY ( "false positive" ) ) OR ( TITLE-ABS-KEY ( sensitivity ) ) OR ( TITLE-ABS-KEY ( specificity ) ) OR ( TITLE-ABS-KEY ( "white coat hypertension" ) ) OR ( TITLE-ABS-KEY ( "mask* hypertension" ) ) OR ( TITLE-ABS-KEY ( "masked hypertension" ) ) OR ( TITLE-ABS-KEY ( "mask hypertension" ) ) OR ( TITLE-ABS-KEY ( "isolate* office" ) ) OR ( TITLE-ABS-KEY ( "isolate* clinic" ) ) ) OR ( TITLE-ABS-KEY ( "diagnostic performance" ) ) )

**The search terms and search strategy for Cochrane Central Register of Controlled Trials**

((((((((((("clinic blood pressure") OR "office blood pressure") OR "conventional blood pressure") OR "clinic BP measurement") OR CBPM) OR "conventional BP monitoring") OR "clinic BP monitoring")) OR ((((("home blood pressure") OR "home monitoring blood pressure") OR "home BP measurement") OR "HBPM") OR "home BP monitoring")))) AND (((((((((((((((((sensitivity) OR specificity) OR "false positive") OR "false negative") OR "true positive") OR "true negative") OR "diagnostic performance"))) OR ((((("white coat") OR "white-coat") OR isolate* AND clinic) OR isolate* AND office) OR "mask hypertension"))) OR "masked hypertension")))))) in Title Abstract Keyword

**The search terms and search strategy used for WHO International Clinical Trials Registry Platform**

((((((((((("clinic blood pressure") OR "office blood pressure") OR "conventional blood pressure") OR "clinic BP measurement") OR CBPM) OR "conventional BP monitoring") OR "clinic BP monitoring")) OR ((((("home blood pressure") OR "home monitoring blood pressure") OR "home BP measurement") OR "HBPM") OR "home BP monitoring")))) AND (((((((((((((((((sensitivity) OR specificity) OR "false positive") OR "false negative") OR "true positive") OR "true negative") OR "diagnostic performance"))) OR ((((("white coat") OR "white-coat") OR isolate* AND clinic) OR isolate* AND office) OR "mask hypertension"))) OR "masked hypertension"))))))

Additional Table 1: Risk of bias assessment based on QUADAS- 2 domains

| **Authors** | | **Risk of bias** | | | | | **Applicability concerns** | | |
| --- | --- | --- | --- | --- | --- | --- | --- | --- | --- |
|  |  | **Subject selection** | **Index test** | | **Reference standard** | **Flow and timing** | **Subject selection** | **Index test** | **Reference standard** |
| **CBPM studies** | | | | | | | | | |
| Brueren M. 1995^1^ | Low | | Low | ? | | ? | Low | Low | Low |
| Hoegholm 1999^2^ | Low | | Low | ? | | High | Low | Low | Low |
| Martinez M. 1999^3^ | Low | | Low | ? | | Low | Low | Low | Low |
| Stergiou G, 2000^4^ | Low | | Low | ? | | Low | Low | Low | Low |
| Gan S 2003^5^ | Low | | Low | ? | | High | Low | Low | Low |
| Calvo-Vargus C. 2003^6^ | Low | | Low | ? | | ? | Low | Low | Low |
| Botomino A. 2004^7^ | High | | Low | ? | | ? | Low | Low | Low |
| Ungar A. 2004^8^ | Low | | Low | ? | | Low | Low | Low | Low |
| Tunckale A. 2004^9^ | High | | Low | ? | | ? | Low | Low | Low |
| Ohkubo T. 2005^10^ | Low | | Low | ? | | High | Low | Low | Low |
| Fagard R. 2007^11^ | Low | | Low | ? | | High | Low | Low | Low |
| Wang G. 2007^12^ | Low | | Low | ? | | High | High | Low | Low |
| Trudel X. 2009^13^ | Low | | Low | ? | | Low | Low | Low | Low |
| Shimbo D. 2009^14^ | High | | ? | Low | | Low | Low | Low | Low |
| Schoenthaler A. 2010^15^ | Low | | Low | ? | | Low | Low | Low | Low |
| Viera A. 2010^16^ | High | | Low | ? | | High | Low | Low | Low |
| Hanninen M. 2010^17^ | Low | | Low | ? | | High | Low | Low | Low |
| Ishikawa J. 2010^18^ | Low | | Low | ? | | High | Low | Low | Low |
| Maseko M.2011^19^ | Low | | Low | ? | | Low | Low | Low | Low |
| Bacaksiz A. 2013^20^ | Low | | Low | ? | | Low | Low | Low | Low |
| Sobrino J. 2013^21^ | ? | | Low | ? | | High | Low | Low | Low |
| Berge H. 2013 ^22^ | Low | | Low | ? | | Low | Low | Low | Low |
| Afsar B. 2013^23^ | Low | | Low | ? | | ? | Low | Low | Low |
| Franklin S. 2013^24^ | Low | | Low | ? | | High | Low | Low | Low |
| Alwan H. 2014^25^ | Low | | Low | ? | | High | Low | Low | Low |
| Larsen T. 2014^26^ | Low | | Low | ? | | High | Low | Low | Low |
| Viera A. 2014^27^ | ? | | Low | ? | | ? | Low | Low | Low |
| Pengkeaw P. 2014^28^ | ? | | Low | ? | | Low | Low | Low | Low |
| Conen D. 2014^29^ | Low | | Low | ? | | High | Low | Low | Low |
| Trachsel L. 2015^30^ | High | | Low | ? | | High | Low | Low | Low |
| Aihasmi K. 2015^31^ | ? | | Low | ? | | ? | Low | Low | Low |
| Zhang L. 2015^32^ | Low | | Low | ? | | ? | Low | Low | Low |
| Mutlu S. 2015 ^33^ | High | | Low | ? | | ? | Low | Low | Low |
| Rhee M. 2015^34^ | Low | | Low | ? | | High | Low | Low | Low |
| Mancia G. 2015^35^ | Low | | Low | ? | | Low | Low | Low | Low |
| Redmond N. 2016^36^ | Low | | Low | ? | | Low | Low | Low | Low |
| Piantanida E. 2016^37^ | Low | | Low | ? | | ? | Low | Low | Low |
| Viera A. 2016^38^ | High | | Low | ? | | Low | Low | Low | Low |
| Scuteri A. 2016^39^ | Low | | ? | ? | | High | Low | Low | Low |
| Booth III J. 2017^40^ | Low | | Low | ? | | ? | Low | Low | Low |
| Anstey D 2017^41^ | ? | | Low | ? | | High | Low | Low | Low |
| Fujita H. 2017^42^ | ? | | Low | ? | | ? | Low | Low | Low |
| Malgarejo J. 2017^43^ | Low | | Low | ? | | Low | Low | Low | Low |
| Ozkan S. 2018^44^ | Low | | Low | ? | | ? | Low | Low | Low |
| Gun T. 2018^45^ | High | | Low | ? | | High | Low | Low | Low |
| Erdogmus S. 2018^46^ | Low | | Low | ? | | ? | Low | Low | Low |
| Sheppard J.P. 2018^47^ | Low | | Low | ? | | ? | Low | Low | Low |
| Bhattarai M. 2019^48^ | ? | | Low | ? | | ? | ? | Low | Low |
| Cai P. 2019^49^ | Low | | Low | ? | | ? | Low | Low | Low |
| Kaul U. 2019^50^ | Low | | Low | ? | | ? | Low | Low | Low |
| Ntineri A. 2019^51^ | Low | | Low | ? | | ? | Low | Low | Low |
| Michaud A. 2019^52^ | Low | | Low | ? | | Low | High | Low | Low |
| Salazar M. R., 2019^53^ | Low | | Low | ? | | Low | Low | Low | Low |
| Trudel X. 2020^54^ | Low | | Low | ? | | ? | Low | Low | Low |
| **HBPM studies** | | | | | | | | | |
| Stergiou G, 2000^4^ | Low | | Low | ? | | Low | Low | Low | Low |
| Hanninen M. 2010^17^ | Low | | Low | ? | | High | Low | Low | Low |
| de Almeida A., 2012^55^ | High | | ? | Low | | Low | Low | Low | Low |
| de Almeida A, 2014^56^ | Low | | ? | Low | | Low | Low | Low | Low |
| Zhang L. 2015^32^ | Low | | Low | ? | | High | Low | Low | Low |
| Park J. 2017^57^ | Low | | Low | ? | | High | Low | Low | Low |
| Rhee M.Y., 2018^58^ | Low | | Low | ? | | Low | Low | Low | Low |
| Ntineri A. 2019^51^ | Low | | Low | ? | | ? | Low | Low | Low |

Additional Table 2. Data used of pooling diagnostic performance of clinic blood pressure measurement compared to ambulatory blood pressure measurement

| **Author year** | **No. of CBPM measures** | **Time ABPM measures** | **ABPM cut-off (mmHg)** | **Interval^a^ (day)** | **N** | **TP** | **TN** | **FP** | **FN** |
| --- | --- | --- | --- | --- | --- | --- | --- | --- | --- |
| Brueren M.1995^1^ | 3 | Daytime | ≥91 (DBP) | - | 94 | 32 | 24 | 22 | 16 |
| Stergiou G.2000^4^ | 5 | Daytime | ≥135/85 | < 7 | 133 | 71 | 27 | 23 | 12 |
| Botomino A. 2004^7^ | 1 | Daytime | ≥135/85 | - | 50 | 3 | 17 | 3 | 5 |
| Calvo-Vargus C. 2003^6^ | 1 | Daytime | ≥135/85 | - | 90 | 41 | 21 | 19 | 9 |
| Ungar A 2004^8^ | 1 | Daytime | ≥135/85 | 1 | 386 | 254 | 35 | 66 | 33 |
| Ohkubo T. 2005 ^10^ | 1 | Daytime | ≥135/85 | - | 1332 | 202 | 739 | 170 | 221 |
| Fagard R.2007^11^ | 1 | Daytime | ≥135/85 | - | 1485 | 143 | 1020 | 146 | 176 |
| Wang G. 2007^12^ | 1 | Daytime | ≥135/85 | - | 694 | 243 | 322 | 54 | 75 |
| Trudel X 2009^13^ | 1 | Daytime | ≥135/85 | - | 2370 | 221 | 1761 | 32 | 356 |
| Hanninen M. 2010^17^ | 4 | Daytime | ≥140/85 | - | 254 | 27 | 191 | 7 | 29 |
| Ishikawa J. 2010^18^ | 1 | 24-hour | ≥130/80 | - | 129 | 31 | 74 | 11 | 13 |
| Maseko M. 2011^19^ | 1 | Daytime | ≥140/85 | < 1 | 689 | 132 | 390 | 124 | 43 |
| Afsar B. 2013^23^ | 1 | Daytime | ≥135/85 | - | 309 | 81 | 85 | 112 | 31 |
| Berge H. 2013 ^22^ | 1 | Daytime | ≥135/85 | < 1 | 62 | 15 | 17 | 11 | 19 |
| Alwan H. 2014^25^ | 1 | Daytime | ≥135/85 | - | 652 | 146 | 386 | 17 | 103 |
| Conen D. 2014^29^ | 1 | 24-hour | ≥130/80 |  | 9550 | 1501 | 5926 | 753 | 1370 |
| Aihasmi K.2015^31^ | - | Daytime | ≥135/85 | - | 150 | 49 | 68 | 21 | 12 |
| Zhang L. 2015^32^ | 3 | Daytime | ≥135/85 | - | 831 | 197 | 287 | 26 | 321 |
| Rhee M. 2015 ^34^ | 1 | 24-hour | ≥130/80 | - | 462 | 100 | 271 | 8 | 83 |
| Mutlu S. 2016 ^33^ | - | 24-hour | ≥130/80 | - | 160 | 84 | 49 | 13 | 15 |
| Scuteri A. 2016^39^ | - | 24-hour | ≥130/80 | - | 2962 | 742 | 1871 | 279 | 63 |
| Melgarejo J. 2017 ^43^ | 1 | 24-hour | ≥130/80 | - | 4997 | 1079 | 2759 | 598 | 561 |
| Fujita H. 2018 ^42^ | - | Daytime | ≥135/85 | - | 198 | 38 | 107 | 43 | 10 |
| Erdogmus S. 2018^46^ | 1 | 24-hour | ≥130/80 | - | 1053 | 394 | 394 | 147 | 118 |
| Sheppard J.P. 2018^47^ | 1 | Daytime | ≥135/85 | - | 268 | 156 | 88 | 16 | 8 |
| Bhattarai M. 2019^48^ | 1 | 24-hour | ≥130/80 | - | 140 | 98 | 10 | 20 | 12 |
| Cai P. 2019^49^ | 1 | 24-hour | ≥130/80 | - | 1056 | 257 | 422 | 132 | 245 |
| Kaul U. 2019^50^ | 1 | 24-hour | ≥130/80 | - | 27472 | 15246 | 3629 | 3304 | 5293 |
| Ntineri A. 2019^51^ | 3 | 24-hour | ≥130/80 | 10-21 | 1971 | 805 | 663 | 185 | 318 |
| Michaud A. 2019^52^ | 1 | 24-hour | ≥130/80 | - | 65 | 20 | 30 | 12 | 3 |
| Trudel X. 2020^54^ | 1 | Daytime | ≥135/85 | 0 | 6733 | 1262 | 4451 | 113 | 907 |

^a^Interval between CBPM and ABPM

ABPM; ambulatory blood pressure monitoring, FP; false positive, FN; false negative, HT; hypertension, TP; true positive, TN; true negative,

Additional Table 3. Subgroup analysis of diagnostic performances of clinic blood pressure measurement comparing with any types of ambulatory blood pressure measurement

| Subgroup | n | Sensitivity%  (95%CI) | Specificity%  (95%CI) | ROC area  (95%CI) | LR+ | DOR  (95%CI) | I^2^ (%)  (95%CI) |
| --- | --- | --- | --- | --- | --- | --- | --- |
| **Risk of bias** |  |  |  |  |  |  |  |
| Low risk | 7 | 73 (60, 83) | 75 (51, 89) | 0.79 (0.72, 0.85) | 2.9 (1.5, 5.3) | 8 (5, 14) | 100 |
| High and unclear risk | 24 | 69 (61, 76) | 82 (75, 88) | 0.82 (0.73, 0.88) | 3.9 (2.7, 5.5) | 10 (7, 16) | 100 |
| **Age (year)** |  |  |  |  |  |  |  |
| <50 | 15 | 68 (59, 77) | 87 (76, 93) | 0.84 (0.80,0.87) | 5.1 (3.0, 8.7) | 14 (8, 24) | 100 (100, 100) |
| >50 | 16 | 71 (62, 79) | 74 (65, 81) | 0.79 (0.61, 0.90) | 2.8 (2.1, 3.6) | 7 (5, 11) | 100 (100, 100) |
| **Male proportion** |  |  |  |  |  |  |  |
| <50% | 14 | 61 (51, 70) | 89 (81, 94) | 0.81 (0.63, 0.91) | 5.8 (3.5, 9.8) | 13 (8, 23) | 100 (100, 100) |
| >50% | 15 | 77 (69, 83) | 71 (61, 79) | 0.80 (0.73, 0.86) | 2.6 (1.9, 3.6) | 8 (5, 13) | 100 (100, 100) |
| **Setting** |  |  |  |  |  |  |  |
| Community | 16 | 61 (53, 68) | 90 (84, 94) | 0.81 (0.63, 0.91) | 6.0 (3.9, 9.3) | 14 (8, 23) | 100 (100, 100) |
| Health care | 15 | 78 (70, 85) | 66 (56, 75) | 0.79 (0.71-0.85) | 2.3 (1.8, 3.0) | 7 (4, 12) | 100 (100, 100) |
| **Number of visits of repeated CBPM** |  |  |  |  |  |  |  |
| 1 visit | 22 | 69 (61, 76) | 81 (71, 88) | 0.80 (0.62, 0.91) | 3.7 (2.5, 5.4) | 10 (6, 15) | 100 (100, 100) |
| 2-5 visits | 9 | 71 (59, 81) | 81 (69, 89) | 0.82 (0.75, 0.88) | 3.7 (2.3, 5.9) | 10 (5, 20) | 99 (99, 100) |
| **Included HT case in subjects** |  |  |  |  |  |  |  |
| No | 6 | 70 (49, 85) | 79 (56, 92) | 0.81 (0.75, 0.86) | 3.3 (1.5, 7.3) | 9 (3, 27) | 100 (99, 100) |
| Yes | 25 | 70 (63, 76) | 81 (73, 88) | 0.81 (0.73, 0.87) | 3.8 (2.7, 5.3) | 10 (7, 15) | 100 (100, 100) |

ABPM; ambulatory blood pressure measurement, CBPM; clinic blood pressure measurement, CI; confidence interval, DOR; diagnostic odds ratio, HT; hypertension, LR+; likelihood ratio positive, ROC; receive operating curve

Additional Table 4. Data used of pooling diagnostic performance of home blood pressure measurement compared to ambulatory blood pressure measurement

| **Authors, year** | **Measurement duration (day)** | **Type of ABPM** | **Cut-off of ABPM for HT diagnosis (mmHg)** | **n** | **TP** | **TN** | **FP** | **FN** |
| --- | --- | --- | --- | --- | --- | --- | --- | --- |
| Stergiou G. 2000^4^ | 6 | Daytime | 135/85 | 133 | 61 | 38 | 22 | 12 |
| Hanninen M. 2010^17^ | 7 | Daytime | 140/85 | 258 | 44 | 186 | 12 | 12 |
| Almeida A., 2012^55^ | 5 | 24-hour | 130/80 | 158 | 31 | 79 | 19 | 29 |
| de Almeida, 2014^56^ | 3 | Daytime | 135/85 | 158 | 41 | 93 | 10 | 14 |
| Zhang L, 2015^32^ | 7 | 24-hour | 130/80 | 1349 | 295 | 280 | 33 | 223 |
| Park J. 2017^57^ | 7 | 24-hour | 130/80 | 256 | 158 | 40 | 11 | 47 |
| Rhee M.Y., 2018^58^ | 7 | 24-hour | 130/80 | 157 | 94 | 24 | 8 | 31 |
| Ntineri A., 2019^51^ | 3 | 24-hour | 130/80 | 1971 | 933 | 641 | 207 | 190 |

ABPM; ambulatory blood pressure measurement, FP; false positive, FN; false negative, HT; hypertension, TP; true positive, TN; true negative

**Additional Table 5.** Data used for pooling proportion of white coat hypertension among positive clinic blood pressure measurement compared with ambulatory blood pressure measurement

| **Authors Year** | **No. of CBPM measures** | **Time ABPM measuring** | **Cut-off of ABPM for diagnosis HT (mmHg)** | **No +ve CBPM** | **TP** | **FP** |
| --- | --- | --- | --- | --- | --- | --- |
| Brueren M.1995^1^ | 3 | Daytime | ≥91 (DBP) | 54 | 32 | 22 |
| Hoegholm A. 1999 ^2^ | 3 | Daytime | ≥135/85 | 420 | 344 | 76 |
| Martinez M.1999 ^3^ | 3 | Daytime | ≥135/85 | 345 | 209 | 136 |
| Stergiou G.2000^4^ | 5 | Daytime | ≥135/85 | 94 | 71 | 23 |
| Gan S. 2003 ^5^ | 2 | 24-hour | ≥135/85 | 118 | 52 | 66 |
| Calvo-Vargus C. 2003^6^ | 1 | Daytime | ≥135/85 | 60 | 41 | 19 |
| Botomino A. 2004^7^ | 1 | Daytime | ≥135/85 | 6 | 3 | 3 |
| Tunckale A. 2004 ^9^ | 3 | Daytime | ≥135/85 | 290 | 167 | 123 |
| Ungar A 2004^8^ | 1 | Daytime | ≥135/85 | 320 | 254 | 66 |
| Ohkubo T. 2005 ^10^ | 1 | Daytime | ≥135/85 | 372 | 202 | 170 |
| Fagard R.2007^11^ | 1 | Daytime | ≥135/85 | 289 | 143 | 146 |
| Wang G. 2007^12^ | 1 | Daytime | ≥135/85 | 297 | 243 | 54 |
| Trudel X 2009^13^ | 1 | Daytime | ≥135/85 | 253 | 221 | 32 |
| Shimbo D. 2009 ^14^ | 2 | Daytime | ≥135/85 | 84 | 75 | 9 |
| Hanninen M. 2010^17^ | 4 | Daytime | ≥140/85 | 34 | 27 | 7 |
| Ishikawa J. 2010^18^ | 1 | 24-hour | ≥130/80 | 42 | 31 | 11 |
| Maseko M. 2011^19^ | 1 | Daytime | ≥140/85 | 256 | 132 | 124 |
| Afsar B. 2013^23^ | 1 | Daytime | ≥135/85 | 193 | 71 | 112 |
| Berge H. 2013 ^22^ | 1 | Daytime | ≥135/85 | 26 | 15 | 11 |
| Alwan H. 2014^25^ | 1 | Daytime | ≥135/85 | 163 | 146 | 17 |
| Pengkeaw P. 2014 ^28^ | 1 | Daytime | ≥135/85 | 31 | 17 | 14 |
| Conen D. 2014^29^ | 1 | 24-hour | ≥130/80 | 2254 | 1501 | 753 |
| Aihasmi K.2015^31^ | - | Daytime | ≥135/85 | 70 | 49 | 21 |
| Zhang L. 2015^32^ | 3 | Daytime | ≥135/85 | 223 | 197 | 26 |
| Rhee M. 2015 ^34^ | 1 | 24-hour | ≥130/80 | 108 | 100 | 8 |
| Mancia G.2015 ^35^ | 2 | 24-hour | ≥130/80 | 1168 | 795 | 373 |
| Mutlu S. 2016 ^33^ | - | 24-hour | ≥130/80 | 96 | 83 | 13 |
| Scuteri A. 2016^39^ | - | 24-hour | ≥130/80 | 805 | 742 | 63 |
| Fujita H. 2018 ^42^ | - | Daytime | ≥135/85 | 81 | 38 | 43 |
| Melgarejo J. 2017 ^43^ | 1 | 24-hour | ≥130/80 | 1677 | 1079 | 598 |
| Erdogmus S. 2018^46^ | 1 | 24-hour | ≥130/80 | 541 | 394 | 147 |
| Sheppard J.P. 2018^47^ | 1 | Daytime | ≥135/85 | 172 | 156 | 16 |
| Bhattarai M. 2019^48^ | 1 | 24-hour | ≥130/80 | 118 | 98 | 20 |
| Cai P. 2019^49^ | 1 | 24-hour | ≥130/80 | 389 | 257 | 132 |
| Kaul U. 2019^50^ | 1 | 24-hour | ≥130/80 | 18550 | 15246 | 3304 |
| Ntineri A. 2019^51^ | 3 | 24-hour | ≥130/80 | 990 | 805 | 185 |
| Michaud A. 2019^52^ | 1 | 24-hour | ≥130/80 | 32 | 20 | 12 |
| Trudel X. 2020^54^ | 1 | Daytime | ≥135/85 | 1375 | 1262 | 113 |

ABPM; ambulatory blood pressure measurement; CBPM; clinic blood pressure measurement; DBP; diastolic blood pressure, FP; false positive, HT; hypertension, TP; true positive

**Additional Table 6.** Data used for pooling proportion of masked hypertension among negative clinic blood pressure measurement compared with ambulatory blood pressure measurement

| **Author Year** | **No. of visit** | **Type of ABPM** | **ABPM cut-off** | **No-negative CBPM** | **TN** | **FN** |
| --- | --- | --- | --- | --- | --- | --- |
| Brueren M.1995^1^ | 3 | Daytime | ≥91 (DBP) | 40 | 24 | 16 |
| Stergiou G.2000^4^ | 5 | Daytime | ≥135/85 | 39 | 27 | 12 |
| Calvo-Vargus C. 2003^6^ | 1 | Daytime | ≥135 (SBP) | 30 | 21 | 9 |
| Botomino A. 2004^7^ | 1 | Daytime | ≥135/85 | 22 | 17 | 5 |
| Ungar A 2004^8^ | 1 | Daytime | ≥135/85 | 66 | 33 | 33 |
| Ohkubo T. 2005 ^10^ | 1 | Daytime | ≥135/85 | 960 | 739 | 221 |
| Wang G. 2007^12^ | 1 | Daytime | ≥135/85 | 397 | 322 | 75 |
| Fagard R.2007^11^ | 1 | Daytime | ≥135/85 | 1096 | 1020 | 76 |
| Trudel X 2009^13^ | 1 | Daytime | ≥135/85 | 2117 | 1761 | 356 |
| Hanninen M. 2010^17^ | 4 | Daytime | ≥140/85 | 220 | 182 | 38 |
| Schoenthaler A. 2010 ^15^ | 1 | Daytime | ≥135/85 | 240 | 190 | 50 |
| Viera A. 2010^16^ | 1 | Daytime | ≥135/85 | 48 | 22 | 26 |
| Ishikawa J. 2010^18^ | 1 | 24-hour | ≥130/80 | 87 | 74 | 13 |
| Maseko M. 2011^19^ | 1 | Daytime | ≥140/85 | 433 | 390 | 43 |
| Bacaksiz A. 2013^20^ | 1 | Daytime | ≥135/85 | 110 | 92 | 18 |
| Afsar B. 2013^23^ | 1 | Daytime | ≥135/85 | 166 | 85 | 81 |
| Sobrino J. 2013^21^ | - | Daytime | ≥135/85 | 485 | 374 | 111 |
| Franklin S. 2013^24^ | 1 | Daytime | ≥135/85 | 5486 | 4455 | 1031 |
| Berge H.2013 ^22^ | 1 | Daytime | ≥135/85 | 26 | 17 | 9 |
| Alwan H. 2014^25^ | 1 | Daytime | ≥135/85 | 489 | 386 | 103 |
| Larsen T. 2014^26^ | 3 | 24-hour | ≥135/85 | 73 | 40 | 33 |
| Viera A.J. 2014^27^ | 1 | 24-hour | ≥130/80 | 294 | 90 | 204 |
| Conen D. 2014^29^ | 1 | 24-hour | ≥130/80 | 7296 | 5926 | 1370 |
| Aihasmi K.2015^31^ | - | Daytime | ≥135/85 | 80 | 68 | 12 |
| Zhang L. 2015^32^ | 3 | Daytime | ≥135/85 | 608 | 263 | 345 |
| Rhee M. 2015 ^34^ | 1 | 24-hour | ≥130/80 | 354 | 271 | 83 |
| Trachsel L. 2015 ^30^ | 1 | 24-hour | ≥130/80 | 87 | 54 | 33 |
| Mutlu S. 2016 ^33^ | - | 24-hour | ≥130/80 | 63 | 49 | 14 |
| Redmond N. 2016^36^ | 1 | Daytime | ≥135/85 | 680 | 493 | 187 |
| Scuteri A. 2016^39^ | - | 24-hour | ≥130/80 | 2150 | 1871 | 279 |
| Piantanida E. 2016^37^ | 1 | 24-hour | ≥130/80 | 50 | 37 | 13 |
| Viera A. 2016^38^ | 1 | 24-hour | ≥130/80 | 350 | 243 | 107 |
| Booth III J. 2017^40^ | 1 | Daytime | ≥135/85 | 695 | 561 | 134 |
| Anstey D. 2017 ^41^ | 1 | Daytime | ≥135/85 | 305 | 221 | 84 |
| Fujita H. 2018 ^42^ | - | Daytime | ≥135/85 | 117 | 107 | 10 |
| Melgarejo J. 2017 ^43^ | 1 | 24-hour | ≥130/80 | 3320 | 2759 | 561 |
| Ozkan S. 2018^44^ | - | 24-hour | ≥130/80 | 157 | 9 | 148 |
| Gun T. 2018^45^ | - | 24-hour | ≥130/80 | 85 | 82 | 3 |
| Erdogmus S. 2018^46^ | 1 | 24-hour | ≥130/80 | 512 | 394 | 118 |
| Sheppard J.P. 2018^47^ | 1 | Daytime | ≥135/85 | 96 | 88 | 8 |
| Salazar M. R., 2019^53^ | 1 | 24-hour | Day: ≥135/85  Night: ≥120/70 | 748 | 522 | 226 |
| Bhattarai M. 2019^48^ | 1 | 24-hour | ≥130/80 | 22 | 10 | 12 |
| Cai P. 2019^49^ | 1 | 24-hour | ≥130/80 | 667 | 422 | 245 |
| Kaul U. 2019^50^ | 1 | 24-hour | ≥130/80 | 8922 | 3629 | 5293 |
| Ntineri A. 2019^51^ | 3 | 24-hour | ≥130/80 | 981 | 663 | 318 |
| Michaud A. 2019^52^ | 1 | 24-hour | ≥130/80 | 33 | 30 | 3 |
| Trudel X. 2020^54^ | 1 | Daytime | ≥135/85 | 5358 | 4451 | 907 |

ABPM; ambulatory blood pressure measurement, CBPM clinic blood pressure measurement, DBP; diastolic blood pressure, FN; false negative, HT; hypertension, TN; true negative

Additional Table 7. Subgroup analysis of pooling proportion of white coat and masked hypertension among positive and negative clinic blood pressure measurement

| **Subgroup** | **%WCHT among positive CBPM** | | | **%MHT among negative CBPM** | | |
| --- | --- | --- | --- | --- | --- | --- |
|  | **N** | **% (95% CI)** | **I^2^ (%)** | **N** | **% (95% CI)** | **I^2^ (%)** |
| **Age (year)** |  |  |  |  |  |  |
| <50 | 18 | 27 (20, 33) | 97.79 | 24 | 25 (22, 29) | 97.11 |
| >50 | 20 | 30 (25, 35) | 97.58 | 23 | 26 (18, 35) | 99.54 |
| **Male proportion** |  |  |  |  |  |  |
| <50% | 17 | 26 (19, 32) | 98.22 | 23 | 26 (22, 30) | 98.39 |
| >50% | 15 | 27 (21, 32) | 96.78 | 17 | 28 (17, 38) | 99.61 |
| **Setting** |  |  |  |  |  |  |
| Community | 18 | 30 (23, 37) | 98.39 | 24 | 22 (19, 25) | 97.19 |
| Health care | 20 | 27 (23, 32) | 96.08 | 23 | 31 (21, 40) | 99.19 |
| **Number of visit of repeated CBPM** |  |  |  |  |  |  |
| 1 visit | 23 | 28 (23, 33) | 98.20 | 34 | 28 (22, 33) | 99.45 |
| 2-3 visits | 11 | 29 (21, 37) | 96.65 | 5 | 38 (25, 51) | 96.38 |
| 4-5 visits | 2 | 23 (16, 31) | 99.58 | 2 | 15 (10, 19) | 99.09 |
| **Included HT case in subjects** |  |  |  |  |  |  |
| No | 8 | 32 (21, 44) | 96.43 | 18 | 27 (22, 33) | 97.42 |
| Yes | 29 | 27 (23, 31) | 97.75 | 29 | 26 (19, 33) | 99.57 |

ABPM; ambulatory blood pressure measurement, CI; confidence interval, CBPM; clinic blood pressure measurement; MHT; masked hypertension, WCHT; white coat hypertension

**Additional Figure 1:** Pooled diagnostic odds ratio, likelihood ratios positive and negative of clinic blood pressure measurement using 24-hour ABPM as the reference standard

**Additional Figure 2:** The hierarchic summary receiver operating characteristic curves plotting the diagnostic performance of clinic blood pressure monitoring and home blood pressure monitoring, when using 24-hour ABPM as the reference standard

**Additional Figure 3.** Pooled diagnostic odds ratio, likelihood ratios positive and negative of clinic blood pressure measurement using daytime ABPM as the reference standard

**Additional Figure 4:** Deeks’s funnel plot asymmetry test of clinic blood pressure monitoring and home blood pressure monitoring studies

**Additional Figure 5:** Pooled diagnostic odds ratio, likelihood ratios positive and negative of home blood pressure measurement using 24-hour ABPM as the reference standard

**References**

1. Brueren MM, Dinant GJ, Schouten BJ, et al. [Hypertension diagnosis by the family physician: measurements according to the NHG-standard (Dutch College of General Practitioners) compared with ambulatory blood pressure determination]. *Nederlands tijdschrift voor geneeskunde* 1995;139(6):278-82. [published Online First: 1995/02/11]

2. Hoegholm A, Kristensen KS, Bang LE, et al. White coat hypertension and blood pressure variability. *American Journal of Hypertension* 1999;12(10 I):966-72. doi: 10.1016/S0895-7061(99)00109-0

3. Martinez MA, Garcia-Puig J, Martin JC, et al. Frequency and determinants of white coat hypertension in mild to moderate hypertension: a primary care-based study. Monitorizacion Ambulatoria de la Presion Arterial (MAPA)-Area 5 Working Group. *American journal of hypertension* 1999;12(3):251-9. [published Online First: 1999/04/07]

4. Stergiou GS, Skeva, II, Baibas NM, et al. Diagnosis of hypertension using home or ambulatory blood pressure monitoring: comparison with the conventional strategy based on repeated clinic blood pressure measurements. *J Hypertens* 2000;18(12):1745-51. doi: 10.1097/00004872-200018120-00007 [published Online First: 2001/01/02]

5. Gan SK, Loh CY, Seet B. Hypertension in young adults--an under-estimated problem. *Singapore Med J* 2003;44(9):448-52. [published Online First: 2004/01/27]

6. Calvo-Vargas C, Padilla-Rios V, Troyo-Sanromán R. Loaned self-measurement equipment model compared with ambulatory blood pressure monitoring. *Blood Pressure Monitoring* 2003;8(2):63-70. doi: 10.1097/00126097-200304000-00002

7. Botomino A, Martina B, Ruf D, et al. White coat effect and white coat hypertension in community pharmacy practice. *Blood Press Monit* 2005;10(1):13-8. doi: 10.1097/00126097-200502000-00004 [published Online First: 2005/02/03]

8. Ungar A, Pepe G, Monami M, et al. Isolated ambulatory hypertension is common in outpatients referred to a hypertension centre. *J Hum Hypertens* 2004;18(12):897-903. doi: 10.1038/sj.jhh.1001756 [published Online First: 2004/07/09]

9. Tunçkale A, Aran SN, Karpuz H, et al. Relationship between insulin resistance and end-organ damage in white coat hypertension. *American Journal of Hypertension* 2004;17(11):1011-16. doi: 10.1016/j.amjhyper.2004.07.002

10. Ohkubo T, Kikuya M, Metoki H, et al. Prognosis of "masked" hypertension and "white-coat" hypertension detected by 24-h ambulatory blood pressure monitoring 10-year follow-up from the Ohasama study. *Journal of the American College of Cardiology* 2005;46(3):508-15. doi: 10.1016/j.jacc.2005.03.070 [published Online First: 2005/08/02]

11. Fagard RH, Van Den Broeke C, De Cort P. Prognostic significance of blood pressure measured in the office, at home and during ambulatory monitoring in older patients in general practice. *Journal of Human Hypertension* 2005;19(10):801-07. doi: 10.1038/sj.jhh.1001903

12. Wang GL, Li Y, Staessen JA, et al. Anthropometric and lifestyle factors associated with white-coat, masked and sustained hypertension in a Chinese population. *J Hypertens* 2007;25(12):2398-405. doi: 10.1097/HJH.0b013e3282efeee7 [published Online First: 2007/11/07]

13. Trudel X, Brisson C, Larocque B, et al. Masked hypertension: different blood pressure measurement methodology and risk factors in a working population. *J Hypertens* 2009;27(8):1560-7. doi: 10.1097/HJH.0b013e32832cb036 [published Online First: 2009/05/16]

14. Shimbo D, Kuruvilla S, Haas D, et al. Preventing misdiagnosis of ambulatory hypertension: algorithm using office and home blood pressures. *J Hypertens* 2009;27(9):1775-83. doi: 10.1097/HJH.0b013e32832db8b9 [published Online First: 2009/06/06]

15. Schoenthaler AM, Schwartz J, Cassells A, et al. Daily interpersonal conflict predicts masked hypertension in an urban sample. *Am J Hypertens* 2010;23(10):1082-8. doi: 10.1038/ajh.2010.141 [published Online First: 2010/07/10]

16. Viera AJ, Hinderliter AL, Kshirsagar AV, et al. Reproducibility of masked hypertension in adults with untreated borderline office blood pressure: comparison of ambulatory and home monitoring. *Am J Hypertens* 2010;23(11):1190-7. doi: 10.1038/ajh.2010.158 [published Online First: 2010/07/31]

17. Hanninen MR, Niiranen TJ, Puukka PJ, et al. Comparison of home and ambulatory blood pressure measurement in the diagnosis of masked hypertension. *J Hypertens* 2010;28(4):709-14. doi: 10.1097/HJH.0b013e3283369faa [published Online First: 2010/01/12]

18. Ishikawa J, Hoshide S, Eguchi K, et al. Masked hypertension defined by ambulatory blood pressure monitoring is associated with an increased serum glucose level and urinary albumin-creatinine ratio. *J Clin Hypertens (Greenwich)* 2010;12(8):578-87. doi: 10.1111/j.1751-7176.2010.00286.x [published Online First: 2010/08/11]

19. Maseko MJ, Woodiwiss AJ, Majane OH, et al. Marked underestimation of blood pressure control with conventional vs. ambulatory measurements in an urban, developing community of African ancestry. *Am J Hypertens* 2011;24(7):789-95. doi: 10.1038/ajh.2011.48 [published Online First: 2011/04/01]

20. Bacaksiz A, Erdogan E, Sonmez O, et al. Ambulatory blood pressure monitoring can unmask hypertension in patients with psoriasis vulgaris. *Medical science monitor : international medical journal of experimental and clinical research* 2013;19:501-9. doi: 10.12659/msm.889197 [published Online First: 2013/06/27]

21. Sobrino J, Domenech M, Camafort M, et al. Prevalence of masked hypertension and associated factors in normotensive healthcare workers. *Blood Press Monit* 2013;18(6):326-31. doi: 10.1097/mbp.0000000000000002 [published Online First: 2013/11/07]

22. Berge HM, Andersen TE, Solberg EE, et al. High ambulatory blood pressure in male professional football players. *British journal of sports medicine* 2013;47(8):521-5. doi: 10.1136/bjsports-2013-092354 [published Online First: 2013/03/19]

23. Afsar B. Comparison of demographic, clinical, and laboratory parameters between patients with sustained normotension, white coat hypertension, masked hypertension, and sustained hypertension. *Journal of cardiology* 2013;61(3):222-6. doi: 10.1016/j.jjcc.2012.11.003 [published Online First: 2013/01/09]

24. Franklin SS, Thijs L, Li Y, et al. Masked hypertension in diabetes mellitus: treatment implications for clinical practice. *Hypertension* 2013;61(5):964-71. doi: 10.1161/hypertensionaha.111.00289 [published Online First: 2013/03/13]

25. Alwan H, Pruijm M, Ponte B, et al. Epidemiology of masked and white-coat hypertension: the family-based SKIPOGH study. *PloS one* 2014;9(3):e92522. doi: 10.1371/journal.pone.0092522 [published Online First: 2014/03/26]

26. Larsen TR, Gelaye A, Waanbah B, et al. Prevalence of masked hypertension in African Americans. *J Clin Hypertens (Greenwich)* 2014;16(11):801-4. doi: 10.1111/jch.12418 [published Online First: 2014/10/21]

27. Viera AJ, Lin FC, Tuttle LA, et al. Levels of office blood pressure and their operating characteristics for detecting masked hypertension based on ambulatory blood pressure monitoring. *Am J Hypertens* 2015;28(1):42-9. doi: 10.1093/ajh/hpu099 [published Online First: 2014/06/06]

28. Pengkeaw P, Suwannakarn S. Prevalence of hypertension in suspected hypertensive patients in Rajavithi Hospital using ambulatory blood pressure monitoring. *Journal of the Medical Association of Thailand = Chotmaihet thangphaet* 2014;97 Suppl 11:S25-30. [published Online First: 2014/12/17]

29. Conen D, Aeschbacher S, Thijs L, et al. Age-specific differences between conventional and ambulatory daytime blood pressure values. *Hypertension* 2014;64(5):1073-9. doi: 10.1161/hypertensionaha.114.03957 [published Online First: 2014/09/04]

30. Trachsel LD, Carlen F, Brugger N, et al. Masked hypertension and cardiac remodeling in middle-aged endurance athletes. *J Hypertens* 2015;33(6):1276-83. doi: 10.1097/hjh.0000000000000558 [published Online First: 2015/02/11]

31. Al-Hashmi K, Al-Busaidi N, Amina B, et al. White coat hypertension and masked hypertension among omani patients attending a tertiary hospital for ambulatory blood pressure monitoring. *Oman Med J* 2015;30(2):90-4. doi: 10.5001/omj.2015.20

32. Zhang L, Li Y, Wei FF, et al. Strategies for classifying patients based on office, home, and ambulatory blood pressure measurement. *Hypertension* 2015;65(6):1258-65. doi: 10.1161/hypertensionaha.114.05038 [published Online First: 2015/04/15]

33. Mutlu S, Sari O, Arslan E, et al. Comparison of ambulatory blood pressure measurement with home, office and pharmacy measurements: is arterial blood pressure measured at pharmacy reliable? *Journal of evaluation in clinical practice* 2016;22(1):40-45. doi: 10.1111/jep.12424 [published Online First: 2015/08/26]

34. Rhee MY, Kim SW, Choi EH, et al. Prevalence of Masked Hypertension: a Population-Based Survey in a Large City by Using 24-Hour Ambulatory Blood Pressure Monitoring. *Korean circulation journal* 2016;46(5):681-87. doi: 10.4070/kcj.2016.46.5.681 [published Online First: 2016/10/11]

35. Mancia G, Facchetti R, Grassi G, et al. Adverse Prognostic Value of Persistent Office Blood Pressure Elevation in White Coat Hypertension. *Hypertension* 2015;66(2):437-44. doi: 10.1161/HYPERTENSIONAHA.115.05367

36. Redmond N, Booth JN, 3rd, Tanner RM, et al. Prevalence of Masked Hypertension and Its Association With Subclinical Cardiovascular Disease in African Americans: Results From the Jackson Heart Study. *Journal of the American Heart Association* 2016;5(3):e002284. doi: 10.1161/jaha.115.002284 [published Online First: 2016/03/31]

37. Piantanida E, Gallo D, Veronesi G, et al. Masked hypertension in newly diagnosed hypothyroidism: a pilot study. *Journal of endocrinological investigation* 2016;39(10):1131-8. doi: 10.1007/s40618-016-0488-7 [published Online First: 2016/05/20]

38. Viera AJ, Lin FC, Tuttle LA, et al. Examination of Several Physiological and Psychosocial Factors Potentially Associated With Masked Hypertension Among Low-Risk Adults. *J Clin Hypertens (Greenwich)* 2016;18(8):784-9. doi: 10.1111/jch.12761 [published Online First: 2015/12/29]

39. Scuteri A, Morrell CH, Orru M, et al. Gender specific profiles of white coat and masked hypertension impacts on arterial structure and function in the SardiNIA study. *International journal of cardiology* 2016;217:92-8. doi: 10.1016/j.ijcard.2016.04.172 [published Online First: 2016/05/15]

40. Booth JN, 3rd, Muntner P, Diaz KM, et al. Evaluation of Criteria to Detect Masked Hypertension. *J Clin Hypertens (Greenwich)* 2016;18(11):1086-94. doi: 10.1111/jch.12830 [published Online First: 2016/11/05]

41. Anstey DE, Booth JN, Abdalla M, et al. Predicted Atherosclerotic Cardiovascular Disease Risk and Masked Hypertension among Blacks in the Jackson Heart Study. *Circulation: Cardiovascular Quality and Outcomes* 2017;10(7) doi: 10.1161/CIRCOUTCOMES.116.003421

42. Fujita H, Matsuoka S, Awazu M. Masked Isolated Nocturnal Hypertension in Children and Young Adults. *Pediatric cardiology* 2018;39(1):66-70. doi: 10.1007/s00246-017-1728-0 [published Online First: 2017/09/28]

43. Melgarejo JD, Maestre GE, Thijs L, et al. Prevalence, Treatment, and Control Rates of Conventional and Ambulatory Hypertension Across 10 Populations in 3 Continents. *Hypertension* 2017;70(1):50-58. doi: 10.1161/hypertensionaha.117.09188 [published Online First: 2017/05/10]

44. Ozkan S, Ata N, Yavuz B. Increased masked hypertension prevalence in patients with obesity. *Clinical and experimental hypertension (New York, NY : 1993)* 2018:1-4. doi: 10.1080/10641963.2018.1431262 [published Online First: 2018/02/09]

45. Gun T, Ozkan S, Yavuz B. Is tinnitus an early voice of masked hypertension? High masked hypertension rate in patients with tinnitus. *Clinical and experimental hypertension (New York, NY : 1993)* 2018:1-4. doi: 10.1080/10641963.2018.1465077 [published Online First: 2018/04/24]

46. Erdogmus S, Kutlay S, Celebi ZK, et al. Clinical Correlates of Ambulatory Blood Pressure Phenotypes at a Tertiary Care Hospital in Turkey. *Kidney & blood pressure research* 2018;43(3):690-700. doi: 10.1159/000489742 [published Online First: 2018/05/16]

47. Sheppard JP, Martin U, Gill P, et al. Prospective external validation of the Predicting Out-of-OFfice Blood Pressure (PROOF-BP) strategy for triaging ambulatory monitoring in the diagnosis and management of hypertension: observational cohort study. *Bmj* 2018;361:k2478. doi: 10.1136/bmj.k2478 [published Online First: 2018/06/29]

48. Bhattarai M, Sainju NK, Bhandari B, et al. Prevalence of white coat hypertension among the patients visiting in a tertiary care center, Kathmandu, Nepal. *Kathmandu University Medical Journal* 2019;17(66):119-22.

49. Cai P, Peng Y, Chen Y, et al. Association of thyroid function with white coat hypertension and sustained hypertension. *J Clin Hypertens (Greenwich)* 2019;21(5):674-83. doi: 10.1111/jch.13536 [published Online First: 2019/04/12]

50. Kaul U, Arambam P, Rao S, et al. Usefulness of ambulatory blood pressure measurement for hypertension management in India: the India ABPM study. *J Hum Hypertens* 2019 doi: 10.1038/s41371-019-0243-6 [published Online First: 2019/09/06]

51. Ntineri A, Niiranen TJ, McManus RJ, et al. Ambulatory versus home blood pressure monitoring: Frequency and determinants of blood pressure difference and diagnostic disagreement. *Journal of Hypertension* 2019;37(10):1974-81. doi: 10.1097/HJH.0000000000002148

52. Michaud A, Lamarre-Cliche M, Cloutier L. Screening for hypertension: An elevated office blood pressure measurement is valuable, adding an automated one is even better. *Blood Pressure Monitoring* 2019;24(3):123-29. doi: 10.1097/MBP.0000000000000382

53. Salazar MR, Espeche WG, Balbin E, et al. Prevalence of isolated nocturnal hypertension according to 2018 European Society of Cardiology and European Society of Hypertension office blood pressure categories. *J Hypertens* 2019 doi: 10.1097/hjh.0000000000002278 [published Online First: 2019/10/05]

54. Trudel X, Brisson C, Gilbert-Ouimet M, et al. Long Working Hours and the Prevalence of Masked and Sustained Hypertension. *Hypertension* 2020;75(2):532-38. doi: 10.1161/hypertensionaha.119.12926 [published Online First: 2019/12/20]

55. Almeida AE, Stein R, Gus M, et al. Improved diagnostic accuracy of a 3-day protocol of home blood pressure monitoring for the diagnosis of arterial hypertension. *Blood pressure monitoring* 2013;18(2):119-26. doi: 10.1097/MBP.0b013e32835ebb18 [published Online First: 2013/02/15]

56. de Almeida AEM, Stein R, Gus M, et al. Relevance to home blood pressure monitoring protocol of blood pressure measurements taken before first-morning micturition and in the afternoon. *Arquivos Brasileiros de Cardiologia* 2014;103(4):338-47. doi: 10.5935/abc.20140139

57. Park JS, Rhee MY, Namgung J, et al. Comparison of Optimal Diagnostic Thresholds of Hypertension With Home Blood Pressure Monitoring and 24-Hour Ambulatory Blood Pressure Monitoring. *Am J Hypertens* 2017;30(12):1170-76. doi: 10.1093/ajh/hpx115 [published Online First: 2017/10/11]

58. Rhee MY, Kim JY, Kim JH, et al. Optimal schedule of home blood-pressure measurements for the diagnosis of hypertension. *Hypertension research : official journal of the Japanese Society of Hypertension* 2018;41(9):738-47. doi: 10.1038/s41440-018-0069-6 [published Online First: 2018/07/07]
